# Surveillance of Travellers: An Additional Tool for Tracking Antimalarial Drug Resistance in Endemic Countries

**DOI:** 10.1371/journal.pone.0077775

**Published:** 2013-10-30

**Authors:** Myriam Gharbi, Jennifer A. Flegg, Bruno Pradines, Ako Berenger, Magatte Ndiaye, Abdoulaye A. Djimdé, Cally Roper, Véronique Hubert, Eric Kendjo, Meera Venkatesan, Philippe Brasseur, Oumar Gaye, André T. Offianan, Louis Penali, Jacques Le Bras, Philippe J. Guérin, Members of the French National Reference Center for Imported Malaria Study

**Affiliations:** 1 Unité Mixte de Recherche 216, Institut de Recherche et de Développement, Paris, France; 2 PRES Sorbonne Paris Cité, Faculté de Pharmacie, Paris, France; 3 WorldWide Antimalarial Resistance Network, Oxford, United Kingdom; 4 Ecole des Hautes Etudes en Santé Publique, Sorbonne Paris Cité, Rennes, France; 5 Centre for Tropical Medicine & Nuffield Department of Clinical Medicine, University of Oxford, Oxford, United Kingdom; 6 Département d’Infectiologie de Terrain, Institut de Recherche Biomédicale des Armées, Marseille, France; 7 Unité de Recherche sur les Maladies Infectieuses et Tropicales Emergentes, Aix Marseille Université, Marseille, France; 8 Centre National de Référence du Paludisme, Marseille, France; 9 Malariology Department, Institut Pasteur de Côte d'Ivoire, Abidjan, Côte d'Ivoire; 10 Service de parasitologie, Faculté de Médecine et Pharmacie Université Cheikh Anta Diop, Dakar, Sénégal; 11 Malaria Research and Training Center & Department of Epidemiology of Parasitic Diseases, Faculty of Pharmacy University of Sciences Techniques and Technologies of Bamako, Bamako, Mali; 12 Pathogen Molecular Biology Department of Infectious Tropical Diseases, London School of Hygiene and Tropical Medicine, London, United Kingdom; 13 Centre National de Référence du Paludisme & Service de Parasitologie Mycologie, CHU Bichat-Claude Bernard APHP, Paris, France; 14 Centre National de Référence du Paludisme and Service de Parasitologie Mycologie, CHU Pitié-Salpétrière APHP, Paris, France; 15 University of Maryland School of Medicine, Baltimore, Maryland, United States of America; 16 UMR 198, Institut de Recherche pour le Développement, Dakar, Sénégal; 17 UMR S 707: Epidemiology Information Systems Modeling, INSERM and Université Pierre et Marie-Curie-Paris6, Paris, France; National University of Singapore, Singapore

## Abstract

**Introduction:**

There are growing concerns about the emergence of resistance to artemisinin-based combination therapies (ACTs). Since the widespread adoption of ACTs, there has been a decrease in the systematic surveillance of antimalarial drug resistance in many malaria-endemic countries. The aim of this work was to test whether data on travellers returning from Africa with malaria could serve as an additional surveillance system of local information sources for the emergence of drug resistance in endemic-countries.

**Methodology:**

Data were collected from travellers with symptomatic *Plasmodium falciparum* malaria returning from Senegal (n = 1,993), Mali (n = 2,372), Cote d’Ivoire (n = 4,778) or Cameroon (n = 3,272) and recorded in the French Malaria Reference Centre during the period 1996–2011. Temporal trends of the proportion of parasite isolates that carried the mutant genotype, *pfcrt* 76T, a marker of resistance to chloroquine (CQ) and *pfdhfr* 108N, a marker of resistance to pyrimethamine, were compared for travellers and within-country surveys that were identified through a literature review in PubMed. The *in vitro* response to CQ was also compared between these two groups for parasites from Senegal.

**Results:**

The trends in the proportion of parasites that carried *pfcrt* 76T, and *pfdhfr* 108N, were compared for parasites from travellers and patients within-country using the slopes of the curves over time; no significant differences in the trends were found for any of the 4 countries. These results were supported by *in vitro* analysis of parasites from the field in Senegal and travellers returning to France, where the trends were also not significantly different.

**Conclusion:**

The results have not shown different trends in resistance between parasites derived from travellers or from parasites within-country. This work highlights the value of an international database of drug responses in travellers as an additional tool to assess the emergence of drug resistance in endemic areas where information is limited.

## Introduction

A decline in artemisinin efficacy has recently been confirmed in several regions in Southeast Asia [1], [2], [3]. Concerns are growing about the potential for this artemisinin resistance to spread to sub-Saharan Africa, as it has previously been described for other antimalarial drugs. Indeed, resistance to chloroquine (CQ) and sulfadoxine-pyrimethamine (SP) emerged relatively quickly after their introduction and subsequently spread from Asia to Africa [4], [5]. Early detection of decreasing drug efficacy and the consequent updating of drug policies are crucial elements in the strategy to prevent the emergence or delay the spread of drug resistance [6], [7]. In recent years, considerable effort has been made to improve epidemiological antimalarial resistance surveillance in countries with limited resources. Therapeutic efficacy studies remain the gold standard for guiding drug policy, as they take into account the complex interactions between the host, parasite and drug [8]. However, many settings in endemic countries lack the financial resources necessary to maintain a sustainable, accurate and reliable antimalarial resistance surveillance system, resulting in gaps in the spatial and temporal available information.

In recent years, globalization and a substantial increase in international travel and population mobility, have provided the potential for the rapid spread of infectious diseases and antimicrobial resistance [9]. More than 900 million international journeys are undertaken annually and this figure has been consistently rising over the years (United Nations World Tourism Organization: UNWTO).

Malaria is endemic in over 100 countries and represents an important infectious disease threat for these nations. Of the 125 million people travelling to malaria endemic countries each year, approximately 10,000 malaria infections were reported worldwide in returning travellers in 2010. Under-reporting is thought to be substantial and, hence, this number may, in reality, exceed 30,000 [10]. In Europe, a 10-fold increase in imported infections was reported from 1970 to 2000 (from 1,500 to about 15,000 cases) before decreasing to about 6,000 cases in 2010 (http://data.euro.who.int/cisid); most of these cases were reported in France or the United Kingdom [11]. Travellers who return from endemic countries infected with malaria often present with low immunity against the parasites and there is no risk of re-infection, so they are a particularly valuable source of information.

**Figure 1:**
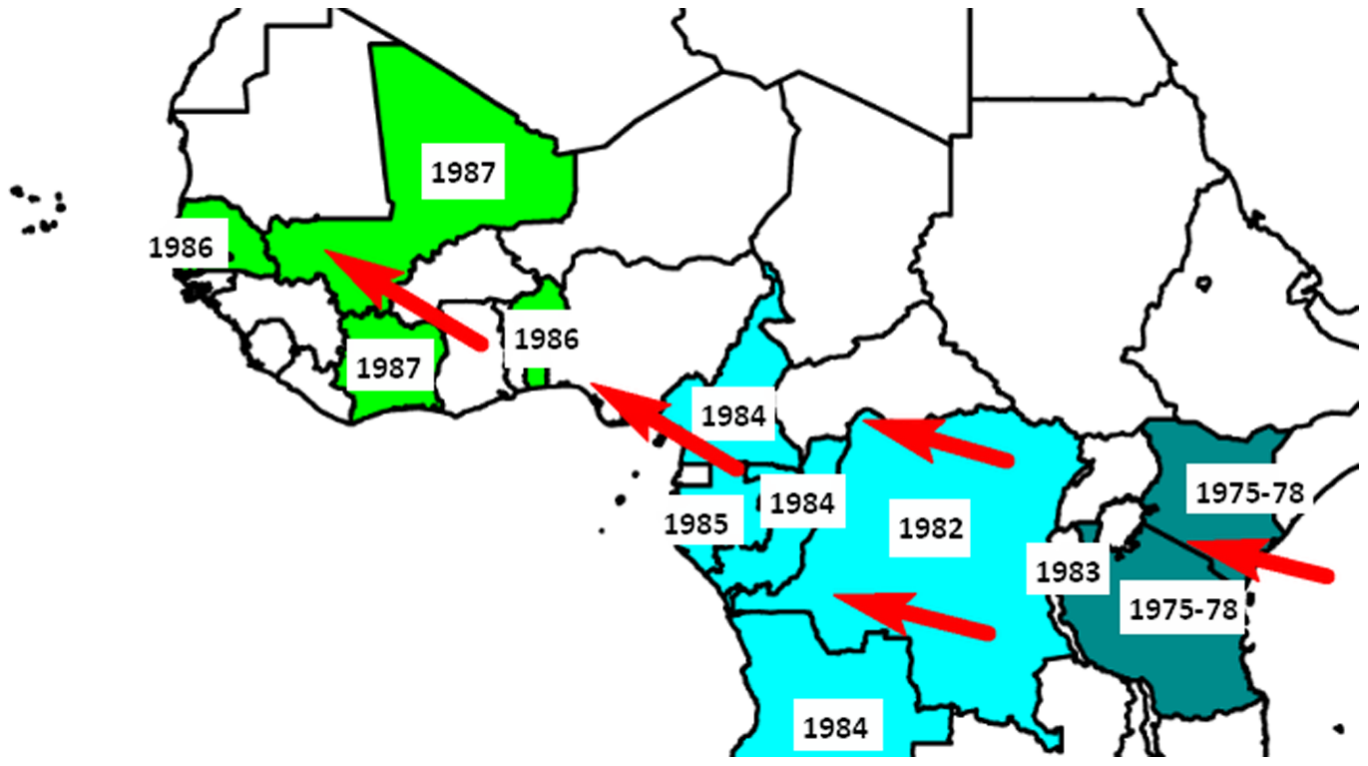
Map of Africa illustrating the emergence of CQ resistance in East, Central and West Africa detected through travellers’ surveillance from the late 1970s to the early 1980s. The dates of detection of index cases are displayed. The red arrows show the spread of antimalarial resistance from East Africa to West Africa.

In fact, historically, the emergence of CQ resistance in Africa was mainly detected through surveillance of travellers (Figure 1, Table 1). The current study was undertaken to test the idea that surveillance of parasites from travellers can be used to accurately assess the evolution of antimalarial drug resistance and provide complementary information to existing monitoring. As a proof of concept, the aim was to compare trends in molecular and *in vitro* markers of drug resistance observed in the imported malaria population with the trends described in field studies.

**Table 1:** First published cases of CQ resistance in Africa through travellers’ surveillance*.

Country	Date case	Date published	Country of detection	Reference
Kenya	1978	1979	Denmark	[29]
East Africa (Kenya-Tanzania)	1975	1979	United States	[30]
Democratic Republic of the Congo (Zaire)	1982	1983	United States	[31]
Burundi	1983	1984	France	[32]
Republic of the Congo	1984	1985	France	[33]
Cameroon	1984	1985	France	[34]
Angola	1984	1984	Denmark	[35]
Gabon	1985	1986	United States	[36]
Benin	1986	1986	France	[37]
Senegal	1986	1987	Sweden	[38]
Cote d’Ivoire	1987	1988	France	[39]
Mali	1987	1988	France	[40]

* The within-country studies which detected the emergence of CQ resistance in other parts of Africa during this time period are not shown here.

## Materials and Methods

### Data Collection

The studies were conducted by the National Reference Centre for Malaria (CNR), Paris, France and investigators from the four endemic countries, in collaboration with the WorldWide Antimalarial Resistance Network (WWARN).

#### Data from travellers

Data were collected from travellers with symptomatic *Plasmodium falciparum* malaria returning from malaria-endemic countries during the period 1996–2011. These cases were reported to the French CNR by one of the 80 hospitals participating in the sentinel network for malaria which covers about half of the cases diagnosed in France. All the travellers included in this study must have visited a malaria-endemic African country in the two months prior to diagnosis and presented with a *P. falciparum* infection biologically confirmed by thin and thick blood smear. Basic demographic, epidemiologic, clinical, and parasitological information as well as response to treatment, previous malaria infection and travel history information were systematically reported. Blood samples were only collected from hospitals which document anti-malarial drug resistance on a systematic basis in all *Plasmodium* positive diagnosis, before treatment, for molecular and *in vitro* analyses.

Molecular markers associated with resistance to CQ and pyrimethamine and CQ susceptibility *in vitro* were the tools used to compare antimalarial drug resistance trends between travellers and field studies.

No informed consent was required for this study as the procedures described here were part of the French national surveillance system of malaria. In 2006, the Commission Nationale de l’Informatique et des Libertés (CNIL), France, approved the electronic-based utilisation of patient-level data collected through the CNR’s questionnaire. We did not receive ethical approval or waiver to perform this secondary research on samples collected as a part of government surveillance for communicable diseases (Loi n° 2004-806 art.16, 21, 9 August 2004, Journal Officiel 11 August 2004). However, ethical aspects have been respected according to the French regulation (article L.1211-2, L.1111-7, L.1413-4 and L.1413-5 Code de la Santé Publique). Also an information note, which explained that the collected blood samples could be used for further research analyses, was provided to the patients who had the possibility to refuse; and the samples were anonymised for this study.

#### Data within-country

A literature review was performed in PubMed for publications on malaria from African endemic countries during the period 1996–2011. The search terms [*country name*+(pfcrt OR chloroquine resistance)] and [*country name*+(DHFR OR sulfadoxine-pyrimethamine resistance OR sulfadoxine pyrimethamine resistance)] were used.

After performing a sample size calculation (see below sample size calculation section), four African countries, Senegal, Mali, Cote d’Ivoire and Cameroon were included in this study. They had sufficiently large numbers of both travellers and field molecular data, from 1996–2011, for a meaningful comparison.

The collaboration of investigators within the four targeted countries, facilitated by WWARN, enabled the collation and standardisation of published field data and the identification and standardisation of unpublished field data. The field studies used for the analyses are summarized in Table 2.

**Table 2:** Summary of the molecular and *in vitro* field studies in the four endemic countries included in the analysis (both published and unpublished).

	Country	Site	Age population	Year of study	Reference
**Molecular marker analysis**					
*Pfcrt* 76	Senegal	Pikine	≥5 ya	2000	[41]
		Pikine	≥18ya	2001	[42]
		Thiadiaye	Pregnant women	2002	[43]
		Dakar	3–65ya	2002	[44]
		Pikine	≥3ya	2004	[45]
		Dakar	All	2004	[46]
		Thies	All	2007	[47]
		Dakar	All	2009–10	[48]
		Central Senegal (3 districts : Mbour, Fatick, Bambey) and Southern Senegal(3 districts : Tambacounda, Velingara, Saraya)	<10 ya	2009–11	[49]
		Dakar	all	2010–2011	Pradines, unpublished data
	Mali	Kolle	<5 ya	2002–03	[50]
		Bougoula-Hameau	>6 mths	2002–04	[51]
		Bancouma, Monteourou, Bandiagara, Faladie, Koulikoro Ba, Sirakoro-meg, Niena, Kolebougou, Markakoungo, Dimbal, Kafana, Siekorole, Toguel, M'pessoba Banamba, N'debougou	all	2002–04	[52]
		Kangaba et kela	>6 mths	2001–03	[53]
		Bandiagara, Faladje Kolle Pongenon	all	2010	Djimdé, unpublished data
	Cote d’Ivoire	Anonkoua-koute (Abidjan), Ayamé, Dabakala	all	2003–08	Ako, unpublished data
		Bonoua and Samo	<5 ya	2005	[54]
		Abidjan (2 districts: Yopougon and Adjamé)	Children	2006	[55]
		Adzope	Children	2007	[56]
	Cameroon	Maroua, Ndop, Bafoussam, Hévécam	<5 ya	2000–01	[57]
		Yaoundé	>12 ya	2000–01	[58]
		Garoua, Yaounde, Mutengene	<5 ya	2004–06	[59]
		Yaoundé, Mfou (suburb of Yaoundé)	All ages	2005–08	[60]
*Pfdhfr* 108	Senegal	Dielmo, Sine Saloum	All	1996–99	[61]
		Pikine, Tambacounda, Thies	≥5 ya	2000–03	[62]
		Dakar	3–65 ya	2002	[44]
		Dakar	<5 ya	2006–08	[63]
		Dakar	all	2009–10	[47]
	Mali	Tieneguebougou	2–12 ya	1996	[64]
		Kidal	All	1999	[65]
		Bandiagara	5–15 ya	2000	[66]
		Kolle	<5 ya	2002–03	[50]
		Bongoula-Hameau	>6 mths	2002–04	[50]
		Kolokani	<5 ya	2006–07	[67]
	Cote d’Ivoire	Yopougon	<5 ya	2000–01	[68]
		Anonkoua-koute (Abidjan), Ayamé, Dabakala		2003–08	Ako, unpublished data
		Bonoua and Samo	<5 y	2005	[54]
		Abidjan (2 districts: Yopougon and Adjamé)	Children	2006	[55]
		Adzope	Children	2007	[56]
	Cameroon	Bertoua, Douala, Eseka, Yaounde	<5 ya	1999	[69]
		Bafoussam, Bertoua, Djoum, Garoua, Hevecam, Manjo, Maroua, Mengang, Ndop, Ngaoundere, Sangmelima, Yaounde	<10 ya	1999–03	[70]
		Dschang, Fontem, Limbe, Nkambe	<10 ya	2002–03	[71]
		Garoua, Mutengene, Yaounde	<5 ya	2004–06	[59]
		Yaounde	≥12 ya	2001–05	[72]
***In vitro*** ** susceptibility test**					
chloroquine	Senegal	Mlomp		1996–98	[73]
		Pikine, Dielmo, NDiop	All	1996	[74]
		Dielmo, NDiop		1997	[75], [76]
		Pikine	≥5 ya	2000	[41]
		Pikine	≥18 ya	2001	[42]
		Dakar		2002	[44]
		Dakar		2009–2010	[77]

### Laboratory Analysis of Parasites

#### Molecular analysis

Two molecular markers, *pfcrt* 76 for CQ resistance and *pfdhfr* 108 for pyrimethamine resistance, were used in this study to compare the trends between travellers and field data. Although the presence of these two markers does not perfectly correlate with treatment failure, each is a good proxy of the intrinsic resistance of the parasite [12], [13]. They are used here as a proof of concept since they have been widely and consistently collected in both field studies and travellers surveillance over the period of interest. Due to the availability of travellers’ data, the time period of 2000–2011 was studied for *pfcrt* 76T, while the time period of 1996–2011 was used for *pfdhfr* 108N.

For molecular analyses of parasites from travellers, DNA was extracted from blood samples of *P. falciparum*, using the QIAamp DNA Mini Kit, Qiagen® before 2008 and the MagNA Pure LC DNA Isolation Kit I, Roche after 2008. PCR and subsequent allele-specific restriction analyses were performed to identify polymorphic codons of interest at the *pfcrt* 76 locus (Lys to Thr) and the *pfdhfr* 108 locus (Ser to Asn) [14].

For field studies, the genotyping methods differed slightly between studies. The detailed method for each study was described in the corresponding publication (see Table 2 for the references to the studies).

Only “pure” *pfcrt* 76T and *pfdhfr* 108N infections among the total number of samples tested were included to improve comparability of the allele prevalence calculated between studies. Indeed, genotyping methods for detecting mixed infections vary in sensitivity across studies. There is not clear evidence that patients living in endemic countries are more likely to carry mixed alleles (mutant and sensitive) than travellers returning from endemic countries as the number of mosquitoes’ bites is not the only factor to consider and the presence of mixed infections is possible after only one bite [15].

#### In vitro assay

For the *in vitro* susceptibility tests, only data from Senegal were analysed in this study because there were sufficient available data for parasites from both the travellers and within-country isolates over the complete period of interest.

For susceptibility tests of parasites from travellers, the following methods were used. The batches of plates were validated on the CQ-susceptible 3D7 reference strain and the CQ-resistant W2 reference strain using the standard 42-hour ^3^H-hypoxanthine uptake inhibition method in controlled atmospheric conditions in the incubator (5% CO_2_, 10% O_2_ and 85% N_2_) [16], [17]. The isotopic microtests were performed, aliquoting 200 µl/well of the suspension of fresh parasitized erythrocytes into 96-well plates pre-dosed with CQ. Radioactivity incorporated by the parasites was measured using a scintillation counter. The CQ susceptibility was calculated as the 50% inhibitory concentration (IC_50_) of CQ of the isolates tested [18], [19]. The drug concentration that inhibited 50% of parasite growth (IC_50_) was estimated by using nonlinear regression to fit an inhibitory sigmoid E_max_ model [20]. The *In Vitro* Analysis and Reporting Tool (IVART) enabled the transformation, standardization and analysis of the data [21].

For within-country surveys, the *in vitro* methods differed between studies and were described in the publications, which are referenced in Table 2. However, the measurement of the drug susceptibility of fresh *P. falciparum* parasites was mainly performed by isotopic assays using the ^3^H-hypoxanthine uptake inhibition method. The *in vitro* CQ susceptibility was determined by a *P. falciparum* Lactate DeHydrogenase (pLDH) ELISA assay in four studies.

### Statistical Analysis

#### Sample size calculation

In order to select eligible countries with enough data per year for significant molecular analysis, a sample size calculation was first performed. The basic comparison of the trends used a simple logistic regression model. The prevalence of isolates from traveller samples that carried a mutant allele (*P_t_*) or from studies on field samples (*P_f_*) was the metric used. In the models: *S.*

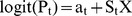


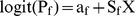
where X is the time covariate and *a* the intercept, the null hypothesis of equal temporal slopes was tested [22]. That is,



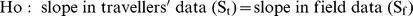



A two sided t-test with a test significance level of α = 0.05, a power of 1−β = 0.80 and an effect size of δ = 0.15 was used. The total sample size required for showing a significant difference between the slopes *S_t_* and *S_f_* was n = 642 isolates for each data type (field and travellers data) per country.

#### Logistic regression

For the molecular analysis, a logistic regression model with time as a linear covariate was fitted to the prevalence of the mutant isolates (separately, for the *pfcrt* 76 and *pfdhfr* 108 data) for the travellers and field studies, for each country. Given the probability of the mutant isolates, the observed number of mutant isolates in each year was assumed to be binomially distributed. The estimated slope of the fitted logistic regression curve for the travellers and field data, the 95% confidence intervals for the slopes and whether the slopes differ significantly from each other are presented (see figures in the results section).

For the *in vitro* susceptibility analysis, a Generalized Linear Model (GLM) with a log-link function was fitted to the travellers and field data for the period 2000–2011. The slopes of the changes in CQ susceptibility for the two datasets were assessed to determine whether they differed significantly from null (0) and whether they differed significantly from each other.

#### Software

All statistical analyses were performed using Stata version 11 for Windows (Stata Corp, College Station, TX, USA) and R version 2.10 (R – project).

## Results

Four African countries had sufficient numbers of field and traveller derived isolates to allow meaningful comparisons between the two populations: Senegal, Mali, Cote d’Ivoire and Cameroon. The characteristics for the field studies are summarised in Table 2 for each publication. A total of 23 studies were included for *pfcrt* 76 analysis over the 2000–2011 period, mainly from Senegal; 21 studies for *pfdhfr* 108 analysis for 1996–2010 and 8 studies from Senegal for the *in vitro* analysis over the same period. The characteristics of the patients differed between studies regarding the population age and the study settings (urban or rural area) but this heterogeneity was observed for the four countries.

The median patient age for travellers experiencing malaria after their return to France was 31 years, with 79% older than 15 and 61% of the travellers had visited friends and relatives in endemic countries for more than one month. Only 38% reported prophylaxis intake during their travel and most patients presented with uncomplicated malaria (95%) (Table 3). No differences among these characteristics were observed among the four countries except for gender; a majority of travellers to Senegal and Mali were male.

**Table 3:** Characteristics of travellers with malaria returning from Senegal, Mali, Cote d’Ivoire and Cameroon and reported in France during the period from 2000 to 2011.

Travellers	Senegal (n = 1,993)*	Mali (n = 2,372)*	Cote d’Ivoire (n = 4,778 )*	Cameroon (n = 3,272)*
Median age (year) [Min-Max]	30 [0–94]	31 [0–76]	30 [0–83]	33 [0–87]
Gender ratio (Male/Female)	2.47	2.20	1.40	1.15
Chemoprophylaxis				
Yes n (%)	746 (38)	959 (41)	1,955 (41)	1,048 (32)
Duration of stay				
≤2 weeks n (%)	218 (13)	152 (8)	457 (12)	439 (16)
2–4 weeks n (%)	356 (21)	361 (18)	1,150 (30)	868 (33)
1–3 months n (%)	699 (41)	928 (48)	1,221 (32)	679 (25)
>3 months n (%)	428 (25)	498 (26)	1,021 (26)	688 (26)
Purpose of travel				
Tourism n (%)	322 (18)	251 (12)	514 (12)	397 (13)
Visit friends and relatives n (%)	1,108 (61)	1,520 (71)	2,482 (58)	1,738 (59)
Severe malaria**				
Yes n (%)	136 (7)	123 (5)	225 (5)	177 (5)

* Numbers may not add to totals because of missing information.

** Severe malaria are cases of imported malaria that fulfilled at least one criteria of the WHO clinical and laboratory classification of severity [78].

Between 2000–2011, 2,874 *P. falciparum* positive isolates were collected from travellers for analysis of the *pfcrt* 76 allele prevalence and 3,351 isolates for analysis of the *pfdhfr* 108 allele prevalence between 1996–2011. Between 1996 and 2011, 305 fresh blood samples were collected from travellers, and tested in Paris or Marseille to measure susceptibility to CQ *in vitro*.

**Figure 2:**
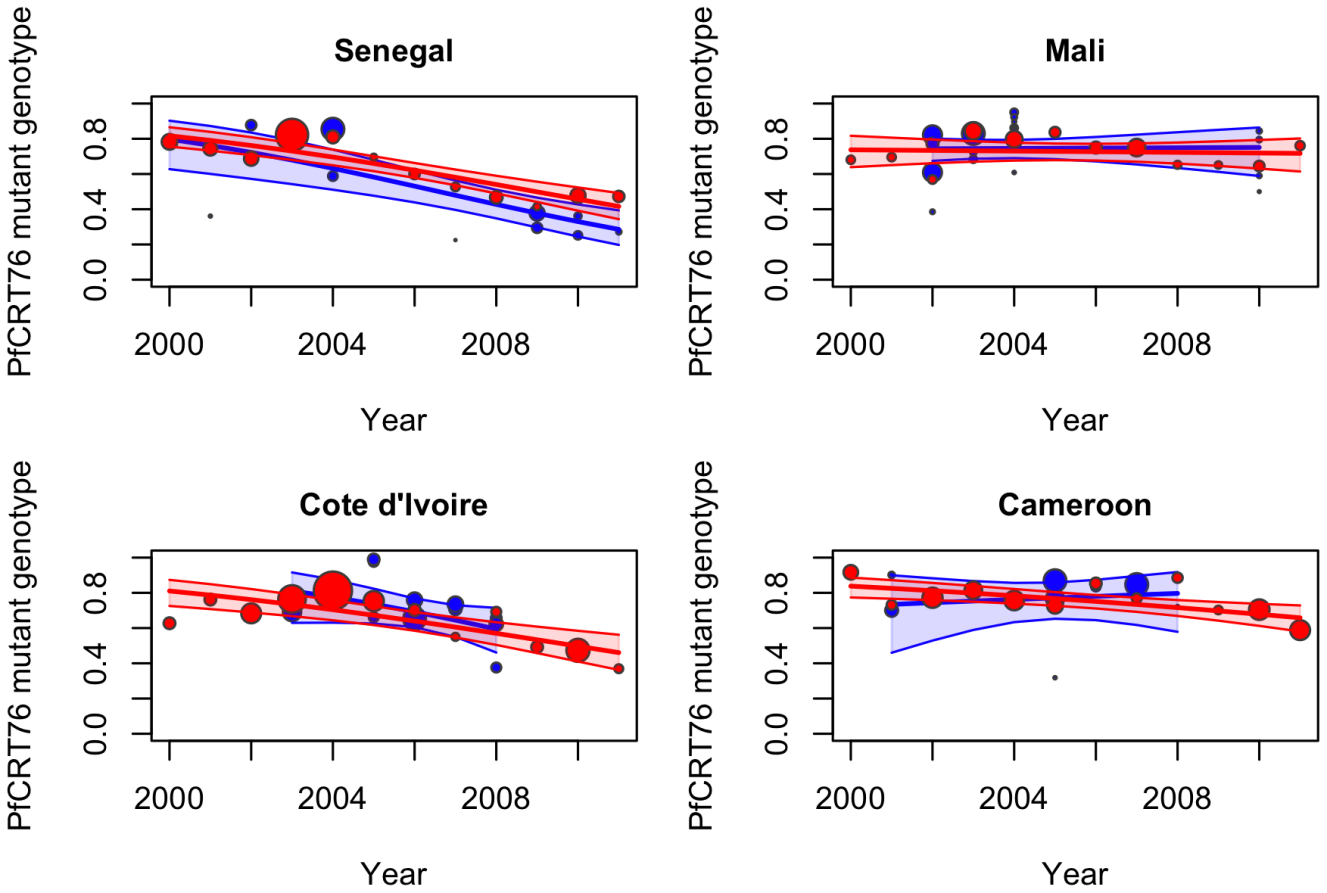
Observed data, fitted model (by logistic regression) and 95% confidence interval (shaded area) for the prevalence of the *pfcrt* 76 mutant isolates from 2000 to 2011 for travellers (red) and field studies (blue) for A-Senegal, B-Mali, C-Cote d’Ivoire and D-Cameroon. Each data point represents the prevalence of resistant isolates per year for travellers’ data and per study for field studies, where the size of the circle is proportional to the number of isolates in the sample.

Figure 2 and Table 4 summarize the temporal trends in the prevalence of the *pfcrt* 76T mutant isolates (associated with CQ resistance) in each of the targeted countries. The prevalence of the *pfcrt* 76T mutant genotype significantly decreased for travellers between 2000 and 2011 in Senegal (*S_t_* = −0.17, p<10^−3^), Cote d’Ivoire (*S_t_* = −0.15, p<10^−3^) and less dramatically in Cameroon (*S_t_* = −0.09, p<10^−3^). However, over that same period, no overall decrease was observed in isolates from Mali (*S_t_* = −0.01, p = 0.72).

**Table 4:** Comparison between travellers and field data for the *pfcrt* 76 and *pfdhfr* 108 molecular markers and for the CQ *in vitro* susceptibility in Senegal.

	Country	Travellers Slope [95% CI*]	Field Study Slope [95% CI]	p-value**
***Pfcrt*** ** 76**	Senegal	−0.167 [−0.219; −0.115]	−0.208 [−0.312; −0.105]	0.575
	Mali	−0.009 [−0.082; 0.063]	0.005 [−0.106; 0.116]	0.885
	Cote d’Ivoire	−0.146 [−0.215; −0.078]	−0.215 [−0.463; 0.032]	0.578
	Cameroon	−0.090 [−0.146; −0.033]	0.050 [−0.220; 0.321]	0.264
***Pfdhfr*** ** 108**	Senegal	0.117 [0.088; 0.147]	0.148 [0.088; 0.209]	0.386
	Mali	0.182 [0.124; 0.240]	0.119 [0.086; 0.152]	0.116
	Cote d’Ivoire	0.083 [0.052; 0.113]	0.132 [−0.025; 0.289]	0.484
	Cameroon	0.213 [0.115; 0.311]	0.130 [−0.155; 0.415]	0.753
**CQ ** ***in vitro*** ** analysis**	Senegal	−0.050 [−0.085; −0.015]	−0.028 [−0.059; 0.002]	0.264

* CI = confidence interval,

** The p-value indicates whether the fitted slopes for travellers data and field studies were significantly different from each other.

After comparing the slopes of the trends for the isolates from travellers (*S_t_*) and from locally studied parasites (*S_f_*), no significant differences were observed between 2000 and 2011 in Senegal (*S_t_* = −0.17 *versus S_f_* = −0.21, p = 0.58), Mali (*S_t_* = −0.01 *versus S_f_* = 0.01, p = 0.89), Cote d’Ivoire (*S_t_* = −0.15 *versus Sf* = −0.22, p = 0.58) and Cameroon (*St* = −0.09 *versus Sf* = 0.05, p = 0.26) (Table 4, Figure 2). After performing a power calculation on the four previous tests, the probabilities of rejecting the null hypothesis, *S_t_* = *S_f_*, when it is false, were between 92 and 96%. These data derived from studies of parasites from returning travellers reflected accurately the trends of the prevalence of molecular markers of CQ resistance that were occurring in the countries in which the travellers acquired their malaria.

**Figure 3:**
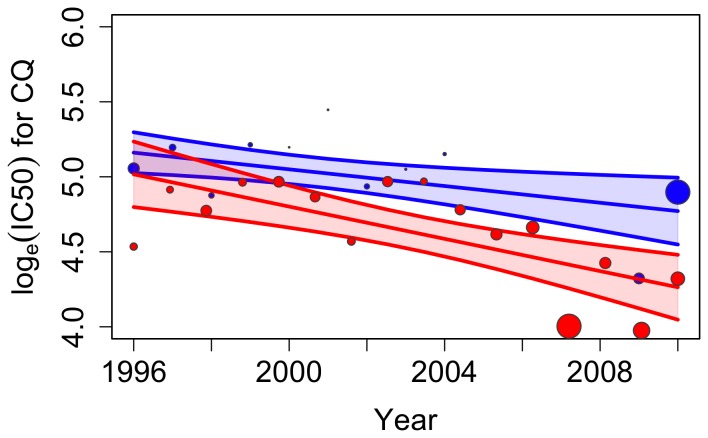
Observed data, fitted model (by Generalized Linear Model) and 95% confidence interval (shaded area) for the *in vitro* CQ response (IC_50_) isolates from 1996 to 2011 for travellers (red) and field studies (blue) from Senegal. Each data point represents the ln (mean IC_50)_ per year for travellers’ data and per study for field studies, where the size of the circle is proportional to the number of isolates in the sample.

Changes in CQ susceptibility were also assessed using the *in vitro* response of isolates. From 1996 to 2011 the geometric mean of the IC_50_ for CQ of the isolates tested *in vitro* decreased in isolates from travellers and those studied in Senegal (Table 4, Figure 3). The geometric means of the IC_50_ values measured for the isolates from travellers were lower than those measured in Senegal. However, the slopes showing the trends did not differ significantly (*S_t_* = −0.05 *versus S_f_* = −0.03, p = 0.26) with a power of 94%. In this case, as well, the data gathered from travellers was an accurate reflection of the trend among parasite populations in the country of origin.

**Figure 4:**
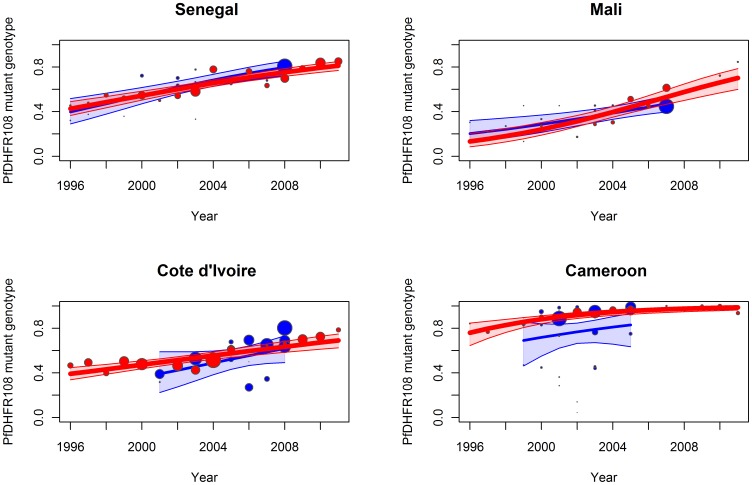
Observed data, fitted model (by logistic regression) and 95% confidence interval (shaded area) for the prevalence of the *pfdhfr* 108 mutant isolates from 1996 to 2011 for travellers (red) and field studies (blue) for A-Senegal, B-Mali, C-Cote d’Ivoire and D-Cameroon. Each data point represents the prevalence of resistant isolates per year for travellers’ data and per study for field studies, where the size of the circle is proportional to the number of isolates in the sample.

The increase of the molecular marker *pfdhfr* 108N has been commonly associated with an increase of pyrimethamine resistance for more than fifteen years [23]. When this parameter was compared between travellers and field-derived isolates, a significant increase in the *pfdhfr* 108N genotype was observed in all 4 countries over the period from 1996–2011: Senegal (*S_t_* = 0.12, p<10^−3^), Mali (*S_t_* = 0.18, p<10^−3^), Cote d’Ivoire (*S_t_* = 0.08, p<10^−3^) and Cameroon (*S_t_* = 0.21, p<10^−3^) (Table 4, Figure 4). For this comparison as well, no significant difference was observed in the trends of the molecular marker *pfdhfr* 108N when data from travellers (*S_t_*) and field-derived (*S_f_*) isolates were compared for samples taken between 1996 and 2011: Senegal (*S_t_* = 0.12 *versus Sf* = 0.15, p = 0.39), Mali (*S_t_* = 0.18 *versus S_f_* = 0.12, p = 0.12), Cote d’Ivoire (*S_t_* = 0.08 *versus S_f_* = 0.13, p = 0.48) and Cameroon (*S_t_* = 0.21 *versus S_f_* = 0.13, p = 0.75) (Table 4, Figure 3). The powers of the four comparative analyses ranged between 91 and 97%.

Thus, all three measures of changes in prevalence of molecular markers and *in vitro* parasites resistance to CQ and pyrimethamine demonstrate that information from parasites imported by travellers was an accurate measure of the changes in parasites within the 4 countries studied.

## Discussion

This study suggests that the surveillance of travellers may be used for monitoring antimalarial drug resistance in endemic countries. The proof of concept was demonstrated using the prevalence of two molecular markers, *pfcrt* 76 and *pfdhfr* 108, and *in vitro* susceptibility for CQ. In this study, no significant difference between the trends of antimalarial drug resistance for travellers’ and field data were observed over more than 10 years. A decrease of the prevalence of the *pfcrt* 76T mutant genotype was observed over a period of 10 years in travellers returning from Senegal, Cote d’Ivoire and Cameroon, whilst this prevalence remains stable in Mali. An increase of mutant genotype isolates for *pfdhfr* 108 was observed in the four countries of West and Central Africa. The *in vitro* CQ susceptibility results supported the molecular results for Senegal. The trend in *pfcrt* 76 is downward while the trend in *pfdhfr* 108 is upward. The fact that screening travellers was able to detect temporal trends in opposite directions strengthens the proof of concept significantly.

Sustainable, reliable and systematic monitoring of drug efficacy is needed for tracking resistance [24]. Monitoring antimalarial drug resistance is based on clinical assessment and biological assays as part of a clinical trial [25]. Since the emergence of resistance to CQ, and then later to SP, capacities to conduct such monitoring in endemic countries have substantially improved, but remain very heterogeneous. In particular regions, human and/or technical resources are limited and, as such, conducting a clinical trial for the purpose of surveillance has competed with other high priorities that Ministries of Health must contend with and has not been systematically conducted.

Previous studies highlight the usefulness of travellers’ surveillance as an early warning detection system for emergence or re-emergence of communicable diseases [26], [27], [28]. Travellers’ surveillance has proven in the past to be an effective early alert system for detecting the emergence of CQ resistance (Table 1).

One strength of using travellers as a sentinel system of resistance is that detection of clinical therapeutic failure due to resistance is facilitated in this non immune population with a low risk of re-infection. Moreover, the French Malaria Reference Centre use standardized methods for prospectively collecting reliable information.

This study does have several limitations. First, due to the complexity of collecting laboratory data systematically, consistently and over a long period of time in both populations, travellers and field studies, only four countries and two molecular markers have been used in this proof of concept. Second, precise information regarding the location of infection within each country could not be collected for *P. falciparum* infected travellers returning from endemic countries. However, travellers did not visit all parts of a country and they were more likely to frequent particular places such as touristic and/or, or business-oriented locations. The reported information was highly dependent on factors such as the areas that were visited, the period of travel, migration history and the political context in endemic areas. Of course, these factors can also impact information on exact locations where patients acquire their infections within the country, as well. Perhaps more importantly, the travellers in this work were not representative of the native population in that their baseline characteristics differ, including age, immune status and parasitemia before treatment. Finally, especially for the field studies, different approaches were used for determining the molecular markers of resistance and the *in vitro* susceptibility for CQ. The heterogeneity between methods is encouraging WWARN to standardise approaches and to develop common tools like IVART [21].

However, these limitations do not diminish the clarity of the outcome presented here. Surveillance of parasites from travellers provided an accurate picture of events occurring in the field. This does not suggest that this approach should replace studies conducted in endemic countries. Rather, information from travellers can be used as an additional surveillance system.

Given the utility, surveillance of travellers can be useful in tracking resistance to ACTs, as well. Currently, only the response to the long-acting partner drugs, can be assessed, but if putative molecular markers are defined, tracking of resistance to the artemisinin component can also be added. The collaboration between Ministries of Health in endemic countries and the malaria reference centres in non-endemic countries for sharing and validating collected information should be reinforced and facilitated.

Due to the length of time between a field study and the publication of results, data collected from imported cases may be available in a more timely manner and, as such, could be used for early alert of emerging resistance. The complexity of the available tools for assessing drug efficacy and monitoring resistance highlights the importance of a standardized and coordinated approach. The follow-up of imported cases in several non-endemic countries should also enable the collaborators to track the evolution of resistance to antimalarial drugs at an international scale and thus provide novel information of value to policy makers.

The goal of this work was to validate the use of international traveller surveillance systems, for detecting the emergence of antimalarial drug resistance and for following resistance trends where local information is not otherwise available and/or sufficient. Easy access to reproducible and standardized data should be implemented. The existing health international, European or American institutions (WHO, European Centre for Disease Prevention and Control (ECDC), US Centres for Disease Control and Prevention (CDC Atlanta) and the different networks for infectious diseases surveillance in travellers (TropNet Europe, EuroTravNet, GeoSentinel) should be used for facilitating the coordination and data sharing between national surveillance systems [26], [27], [28].

## Conclusions

This study has not shown different trends in antimalarial drug resistance between travellers and field studies. An international travellers’ database can be used as an additional surveillance system to assess and monitor the emergence of drug resistance in endemic areas where information is limited.
